# A Framework for Automatic Clustering of EHR Messages Using a Spatial Clustering Approach

**DOI:** 10.3390/healthcare11030390

**Published:** 2023-01-30

**Authors:** Muhammad Ayaz, Muhammad Fermi Pasha, Tham Yu Le, Tahani Jaser Alahmadi, Nik Nailah Binti Abdullah, Zaid Ali Alhababi

**Affiliations:** 1 Malaysia School of Information Technology, Monash University, Jalan Lagoon Selatan Bandar Sunway, Subang Jaya 47500, Selangor, Malaysia; 2 Department of Information System, College of Computer and Information Sciences, Princess Nourah bint Abdulrahman University, Riyadh 11671, Saudi Arabia; 3 Riyadh First Health Cluster, Ministry of Health, Riyadh 11622, Saudi Arabia

**Keywords:** FHIR, healthcare, EHR, DBSCAN, clustering, machine learning

## Abstract

Although Health Level Seven (HL 7) message standards (v2, v3, Clinical Document Architecture (CDA)) have been commonly adopted, there are still issues associated with them, especially the semantic interoperability issues and lack of support for smart devices (e.g., smartphones, fitness trackers, and smartwatches), etc. In addition, healthcare organizations in many countries are still using proprietary electronic health record (EHR) message formats, making it challenging to convert to other data formats—particularly the latest HL7 Fast Health Interoperability Resources (FHIR) data standard. The FHIR is based on modern web technologies such as HTTP, XML, and JSON and would be capable of overcoming the shortcomings of the previous standards and supporting modern smart devices. Therefore, the FHIR standard could help the healthcare industry to avail the latest technologies benefits and improve data interoperability. The data representation and mapping from the legacy data standards (i.e., HL7 v2 and EHR) to the FHIR is necessary for the healthcare sector. However, direct data mapping or conversion from the traditional data standards to the FHIR data standard is challenging because of the nature and formats of the data. Therefore, in this article, we propose a framework that aims to convert proprietary EHR messages into the HL7 v2 format and apply an unsupervised clustering approach using the DBSCAN (density-based spatial clustering of applications with noise) algorithm to automatically group a variety of these HL7 v2 messages regardless of their semantic origins. The proposed framework’s implementation lays the groundwork to provide a generic mapping model with multi-point and multi-format data conversion input into the FHIR. Our experimental results show the proposed framework’s ability to automatically cluster various HL7 v2 message formats and provide analytic insight behind them.

## 1. Introduction

The opportunity to improve the quality of healthcare through interoperability and healthcare IT has recently come to the attention of both the public and private sectors [1,2,3]. Some of these e-heath technologies include electronic medical records (EMRs), electronic health records (EHRs), personal health records (PHRs), and health information exchanges [4,5]. As healthcare systems are increasingly adopting health information technology, a massive volume of clinical data are being collected [6]. These data are stored in EHRs or PHRs of multiple formats, such as in simple database tables, HL7 v2 format, or any other format. These records number in the millions and have become extremely complex. Moreover, any health information system’s stored data are divergent in nature. 

One of the ultimate goals of using health information technology is to evaluate and provide information and benefits to both patients and providers with regard to the most appropriate treatment options based on factors such as cost, benefits, quality, and risk [7,8]. Data standards and interoperability are currently a bottleneck for the smooth exchange and use of data to reap the maximum benefits of health IT. To share health information, interoperability across software from many vendors is crucial [9]. A fundamental requirement for interoperability is the need for both data and message standards. Data interoperability in healthcare information systems and the lack of data standards have been core problems in the healthcare sector for decades. Interoperability problems in the healthcare industry include a gap in data standards, the use of multiple data standards in single organizations, several overlapping standards, etc. [6]. 

There are many key challenges in the processing and exchange of healthcare data that traditional software cannot solve. The first key challenge is that the patient may receive their care from multiple healthcare organizations at different locations, leading to the availability of the patient’s information in various EHRs in different formats. Therefore, there is a need to aggregate the availability of these data in a heterogeneous healthcare environment. The healthcare interchange data help to ensure communication among different EHRs, allowing the exchange of patient information to support the quality of patient treatment. This can be achieved with the development of a common standard that could be capable of exchanging patient details among clinicians at different healthcare providers. The communication of information from one EHR format to another is possible through interoperability [10]. However, numerous challenges exist due to the use of multiple data standards in healthcare organizations, such as each EHR using different clinical terminologies, technical specifications, functional capabilities, etc. [11,12]. These differences make it difficult to create a single standard interoperability format for data sharing. Other challenges include (1) lack of consistency when identifying patients in different EHRs; (2) inability to measure, analyze, and bring improvement in interoperability between healthcare systems; and (3) communication difficulties between different EHRs without a common standard.

The second challenge is that the collaboration among various healthcare organizations has increased over time, and these organizations have various EHRs using different data standards. Clinical information is being increased over time in the healthcare databases [13,14] after being collected from various EHRs. Because patient information is spread across various organizations, the data exchange is important, but the process of exchanging data in these organizations is also difficult due to the use of various formats. Therefore, it becomes difficult for such traditional software to choose new types of variables from the dataset for data selection, because there may be new types of variables added in the dataset. For example, in a typical dataset, there are three types of test result data stored in the laboratory information systems. The traditional model uses one type of variable for this data processing. However, in the event that the number of test result types increases, the traditional software will be unable to recognize and select the variable’s type for the new test result type, because the traditional software operates on unintelligent algorithms and predefined variables [15,16]. Therefore, solving this problem using traditional software is also difficult. 

The third challenge is that health information systems have massive amounts of data, and the most challenging task is how best to organize and manage these data. A large portion of these data are currently unstructured in nature, and the key reason is that we record them in various formats (e.g., HL7 v2, databases). Managing this vast volume of unstructured data through traditional software or techniques is difficult [17,18,19]. 

Thus, we can conclude that the healthcare industry has numerous data processing and exchange issues, such as patients’ clinical data being stored in multiple formats, as well as the divergent nature of health information system data. 

Machine learning techniques allow us to address most of the abovementioned healthcare issues and find better solutions [20,21]. Various studies have used machine learning techniques to resolve EHR data issues. For example, machine learning methods have been used to identify health outcomes from EHR data, such as laboratory test results and diagnosis data stored in unstructured formats [22]. Similarly, machine learning methods have also been used to analyze large datasets of clinical data to predict patients’ future suicidal behavior [23]. Moreover, various machine learning methods have been used to process and handle large datasets in healthcare [14,24] and many other fields. However, none of the existing studies has discussed analyzing the HL7 v2 dataset.

Unsupervised machine learning clustering techniques play a significant role in the healthcare industry, and many researchers have used these techniques to cluster simple healthcare EHR datasets. For example, [25] used unsupervised machine learning techniques to explore a subset of EHRs from patient questionnaires. Similarly, [26] used various unsupervised machine learning techniques to identify subgroups of high-cost patients in a healthcare dataset. However, there is a lack of studies using unsupervised clustering techniques on the HL7 v2 dataset, which is one of the core requirements of the healthcare industry because healthcare organizations store patient data in HL7 v2 format, and they need to cluster these data before exchanging them between various EHRs using different data standards. Thus, there is a clear gap in the literature, and we need machine learning (ML) methods or techniques to develop a model to cluster data stored in HL7 v2 format in order to facilitate the data exchange mechanism among different EHRs. 

In this study, we propose a comprehensive framework that aims to convert unstructured EHR data into HL7 v2 format and then apply an unsupervised clustering technique using the DBSCAN algorithm to automatically group various HL7 v2 messages, regardless of their semantic origin. We selected the DBSCAN (density-based spatial clustering of applications with noise) algorithm for our data clustering because it is specially designed to discover the clusters and noises in large datasets. Additionally, it also provides the analytic insight behind them. The proposed framework’s implementation lays the groundwork for a generic mapping model with multi-point and multi-format data conversion input into the FHIR. This is the first step towards mapping HL7 v2 messages into FHIR resources. The proposed model can retrieve the clinical data from the dataset in HL7 v2 format and then apply unsupervised machine learning clustering techniques to cluster these data. The generated cluster data would be ready for exchange across multiple systems. 

Many data standards are used in the healthcare industry for medical data exchanges, but these standards have some issues. For example, the IEEE medical device communication standards are based on an object-oriented systems management paradigm, which affects data integrity and is complicated to implement [27]. The openEHR specification is highly complex and hierarchical, due to which its performance always suffers [28]. ISO 13606’s EHR interoperability has not supported the implementation of messaging components, and its implementation is quite difficult [29]. At the same time, SNOMED CT and LOINC represent the comprehensive clinical reference terminology available in the medical world [30]. 

Therefore, we chose the FHIR as an interoperability standard for future work in this study. Because the FHIR is considered to be the most popular data standard in the healthcare industry, it supports more data formats—such as RESTful API, messages, documents, and services—as an interoperability paradigm. It simplifies software integrations and interoperability in the healthcare industry. Furthermore, it uses JavaScript object notation and XML structures for data exchange and resource serialization. Therefore, it simplifies implementation without sacrificing integrity. Moreover, it enables the developers to create standardized browser applications that let the user access clinical data from any healthcare system, regardless of the operating systems and devices employed by that healthcare system. Additionally, it supports using smart devices—such as smartphones, fitness trackers, and smartwatches—in the healthcare industry. Therefore, our central insight was to develop a framework that directly maps to Fast Health Interoperability Resources (FHIR). 

The rest of this manuscript is organized as follows: Section 2 (Related Work) describes the related work, and Section 3 (HL7 v2 Messages Machine Learning DBSCAN Clustering Model) describes the framework architecture, methodology, and HL7 v2 dataset preparation. Section 4 (Methods) describes the algorithms’ development and analysis, data representation and processing, and the required outcomes. Section 5 (Experiments, Evaluation Measures, and Results) discusses the experimental setup and results. Finally, Section 6 (Conclusions) analyzes the conclusions and future plans.

## 2. Related Work

Machine learning approaches and techniques have been widely applied within the context of healthcare. However, we attempt to highlight only those relevant works that are related to machine learning data-clustering techniques. For a long time, machine learning clustering approaches or techniques have been used in the healthcare sector to predict and diagnose diseases. As a result, they provide fast, cheap, and reliable healthcare delivery to patients. Innumerable studies have been conducted using machine learning data-clustering techniques in healthcare. 

The study of Jabel et al. [31] applied clustering techniques to a heart disease dataset and compared their performance. They evaluated the performance of three clustering algorithms and found that the CLARA (Clustering LARge Applications, Kaufmann and Rousseeuw) clustering technique showed better results than the PAM (Partitioning Around Medoids) and k-means algorithms. Their experiments were limited to the CLARA, PAM, and k-means algorithms and ignored other clustering algorithms, such as density-based clustering and hierarchical algorithms. Nithya et al. [32] extended their work and included other clustering techniques, such as density-based clustering, hierarchical clustering, and k-means clustering techniques. They compared the performance of these three algorithms and claimed that the k-means algorithm gave better results than the other algorithms. The study of Bruno G. et al. [33] proposed a data mining approach based on a density-based clustering algorithm. They used a new integrated distance measure approach for patient clustering, aiming to discover a well-separated group of diabetes patients with the same profile (i.e., gender and age) and examination history. Their results showed that their approach was productive in discovering groups of patients with the same examination history. Similarly, Paul et al. [34] used the k-means clustering algorithm for medical data. They argued that their algorithm could handle both discrete and continuous data. Moreover, the study of Belciug et al. [35] used agglomerative hierarchical clustering algorithms to group patients according to their length of stay in the hospital. This model can help hospital management in the planning and decision-making process. Additionally, the study of Belciug et al. [36] evaluated the performance of three clustering algorithms (self-organizing map, k-means algorithm, and cluster network) using the WRBC (Wisconsin Recurrence Breast Cancer) dataset. Their experimental results showed that the cluster network had higher performance than the other two algorithms.

The study of C. Lopeza et al. [37] used machine learning clustering techniques on clinical data and obtained good results. They discovered patient clusters, and the results were based on genetic signatures. Similarly, J. Yan et al. [26] applied various clustering algorithms to patients’ data and compared their results. They used clustering and sub-clustering methods on patients’ data. Furthermore, [38] discussed the use of unsupervised machine learning clustering methods on datasets of Alzheimer’s disease. It also explained the role of clustering algorithms in treating Alzheimer’s disease. In their study, H. Estiri et al. [39] applied an unsupervised clustering-based detection technique for detecting implausible observations in EHR data. They produced their results based on the hypothesis that when there are a huge number of observations in the EHR dataset, the implausible records should be parsed. They produced the results with extraordinary specificity and high sensitivity. Andrew Shea [40] applied unsupervised clustering techniques on an EHR dataset to address various challenges in the patients’ EHR data. After obtaining clusters, the author conducted high-resolution analysis by examining the most frequent phenotypes within each cluster. The study [25] used well-known k-means unsupervised machine learning techniques to explore a subset of EHRs from patient questionnaires. The results showed that natural groupings most likely existed in the dataset. H. Zong et al. [41] applied machine learning clustering techniques to cluster a dataset. In this dataset, every record contained patients’ demographic information and correlated diagnosis codes. They produced clustering results that had patients’ demographic information and diagnosis codes. 

In [42], the researchers applied a machine learning clustering algorithm to handle the clustering of vertically partitioned data among various healthcare organizations or data providers. The study of T. Aldhyani et al. [43] proposed a k-means clustering algorithm to identify the ambiguity in chronic disease datasets to improve the system performance. Similarly, M. Elbattah et al. [44] used supervised and unsupervised machine learning techniques to support decision-making concerning elderly healthcare in Ireland. They also used the k-means clustering algorithm to group the elderly patients based on various factors, such as length of stay, age similarity, elapsed time to surgery, etc. A. Alsayat et al. [45] used various machine learning clustering algorithms to cluster healthcare data for knowledge discovery. Additionally, the study of G. Ogbuabor [46] analyzed various clustering techniques using healthcare datasets and compared their results. They particularly focused on the results of DBSCAN and k-means algorithms. 

The study of E. Bose et al. [47] used unsupervised machine learning algorithms to identify subgroups of heart failure patients in various healthcare datasets. They also examined inter-cluster differences for patient characteristics related to symptoms, medications, medical history, psychosocial assessments, etc. Similarly, S. P. Singh et al. [48] employed an agglomerative hierarchical clustering algorithm on a healthcare dataset to identify clusters within the patient population. The algorithm grouped the dataset into convenient, distinct clusters. Moreover, M. Ambigavathi et al. [49] analyzed the use of various machine learning clustering algorithms on mixed healthcare data. Moreover, in [14], the researchers discussed the processing, application, and handling of healthcare-related big data using various clustering techniques and compared the efficiency of these techniques. 

Various clustering algorithms have been used for healthcare data clustering. However, the results of the DBSCAN algorithm look impressive. Many studies have used the DBSCAN algorithm for healthcare data clustering. The study of W. Hurst et al. [50] used the DBSCAN algorithm for healthcare data clustering analysis. This algorithm generated excellent results for identifying anomalous behaviors within electronic personal record (EPR) datasets. Similarly, Pasin et al. [51] applied the DBSCAN algorithm for health data clustering and compared their results with those of other clustering algorithms. The results showed that the DBSCAN algorithm was better than the other clustering algorithms. Moreover, the study of M. E. Celebi et al. [52] applied the DBSCAN algorithm to a clinical dataset to identify homogeneous color regions in biomedical images using clustering techniques. J. Hou and H. Gao [53] also employed the DBSCAN algorithm on biomedical image data. However, this algorithm cannot be applied to flat or other clinical data. 

These works show the significant effects of machine learning models in the healthcare domain. However, all of these models were developed on datasets with flat data or data in a simple electronic health record (EHR) format and, thus, worked through simple algorithms. However, in healthcare organizations, the clinical data are stored in HL7 v2 format, but no machine learning model developed on the HL7 v2 dataset to generate optimized results has yet been reported in the literature. Therefore, in this study, we propose a model to go one step further than the previous works and develop an unsupervised machine learning model on the HL7 v2 dataset (rather than a flat dataset or simple EHR format) that supports the exchange of clinical data across multiple systems in any healthcare organization. The proposed model can retrieve the clinical data in HL7 v2 format and then cluster these data. The generated cluster data would be ready for exchange across multiple systems in healthcare organizations.

## 3. HL7 V2 Messages Machine Learning DBSCAN Clustering Model

This section describes the proposed framework architecture, methodology, and HL7 v2 dataset preparation in detail.

### 3.1. Framework Architecture

**Figure 1:**
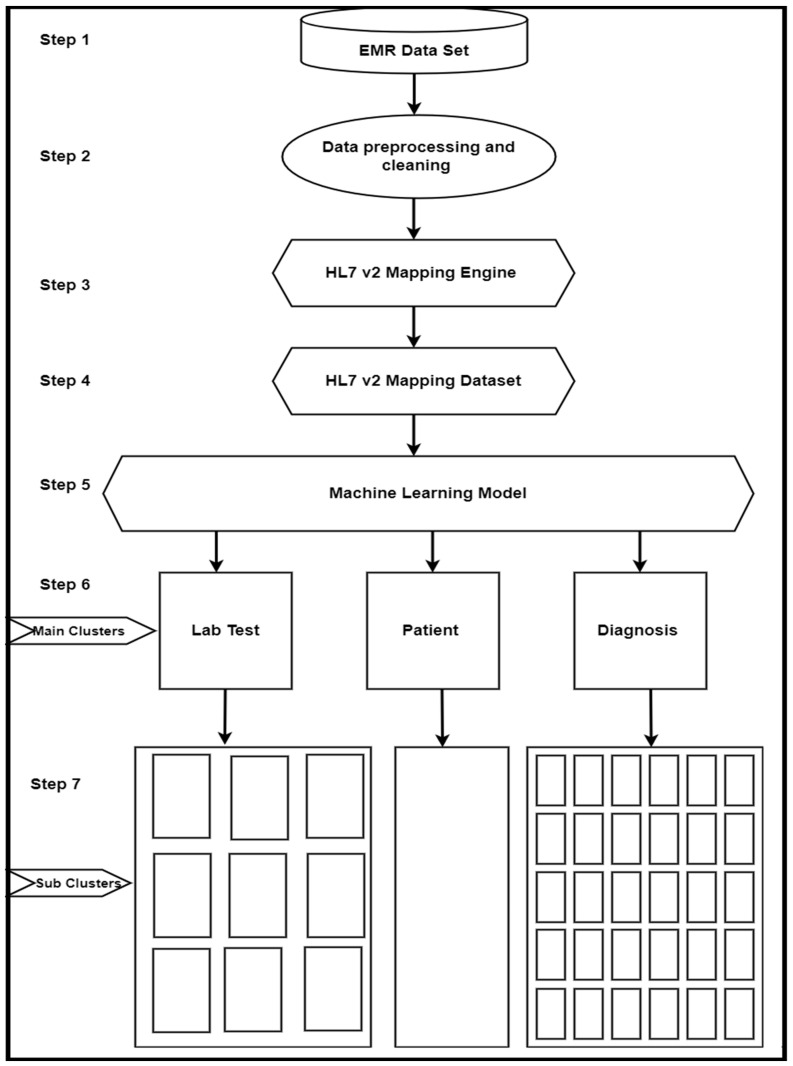
Framework architecture.

The framework proposed in this article is structured in several steps. Its flow is represented in Figure 1. The first step represents a simple EMR dataset with the data that we used for our framework validation. The second step was to perform a preprocessing operation on the raw data, while the third step was to prepare the HL7 v2 dataset. These steps are described in detail in the remainder of this section. It should be noted here that no machine learning approach was applied in these steps.

On the other hand, in steps 4 and 5, we applied a machine learning clustering approach to the HL7 v2 dataset that we prepared in our previous steps. In these steps, we identified the various clusters of our dataset. The details of this work are discussed in Section 4 and Section 5. 

### 3.2. Methodology

The study is based on the following methodology: 

#### 3.2.1. Exploring the Dataset

This step involved searching for a publically available free dataset. We searched many datasets used in the healthcare domain—for example, the Pima Indian Diabetes Dataset (PIDD) [54], Uniform Hospital Discharge Data Set (UHDDS), Uniform Ambulatory Care Data Set (UACDS), Minimum Data Set for long-term care (MDS), Outcomes and Assessment Information Set (OASIS), Data Elements for Emergency Department Systems (DEEDS), Health Plan Employer Data and Information Set (HEDIS), etc. [55]. However, these datasets are used for specific purposes and are not popular among healthcare researchers and industries. We needed a dataset with a complete set of data used in healthcare settings. Therefore, we found the famous MIMIC-III dataset [56], developed by the Massachusetts Institute of Technology (MIT) Laboratory, which provides the complete clinical data used in various healthcare settings.

Furthermore, this database was populated with data that had been collected during the patients’ routine hospital care during the mentioned period. Therefore, real patients’ data were used, such as patients’ clinical information, including demographic information, laboratory test results, imaging reports, caregiver notes, medication, procedures, mortality, etc. Additionally, this dataset is widely used by researchers working in the healthcare domain, including in academia and industry. Therefore, we used this dataset in this work.

#### 3.2.2. Preprocessing Data

This step is related to how to deal with data before input to the framework. This step further involves the following sub-steps:

(a) Data Extraction

This sub-step is related to how to extract the required data from the dataset. To prepare and test our model, we extracted data from the following tables: Patient (patient demographic information), Lab (patient laboratory test results), and Admission Diagnosis (patients’ admitted diagnosis information).

(b) Data Cleaning

Data cleaning entails detecting and correcting any dataset’s incorrect and corrupt data elements. Every dataset has inaccurate data elements, which must be corrected before further processing. Therefore, we cleaned our dataset before training our model on this dataset. 

#### 3.2.3. Dataset Preparation 

We needed the dataset to be in the HL7 v2 message format for our framework validation, but the data that we retrieved from the MIMIC-III database were in a simple EHR format stored in database tables. Therefore, first, we converted these data into the HL7 v2 message format and prepared our dataset before validating our framework on this dataset.

#### 3.2.4. Algorithm Development

This step is related to exploring, developing, and analyzing the algorithm. In this step, we explored various machine learning algorithms, such as k-means clustering, KNN (k-nearest neighbors), hierarchal clustering, etc., which could be suitable for our dataset. However, these algorithms have some issues. For example, the k-means clustering algorithm has issues when clustering data where the clusters are of varying sizes and densities. Furthermore, KNN (k-nearest neighbors) has an issue in picking the correct value of k. Similarly, the hierarchal clustering algorithm has issues with providing many arbitrary decisions and the best solution. Therefore, we chose the DBSCAN algorithm for our data clustering because the DBSCAN has the following capabilities:

1. Handling clusters of multiple sizes and structures (k-mean).
2. Picking the correct value of k for clustering.
3. Providing the best solutions.

This step is discussed in more detail in Section 4. 

#### 3.2.5. Implementation, Experiments, and Results

This step is related to implementing the algorithms, performing various experiments, and generating the results. In this step, we used the Mirth engine tool and the Python programming language to implement our algorithms and test our prototype. We displayed the results of our prototype. This step is discussed in more detail in Section 5. 

### 3.3. Methodology Details 

In this section, we discuss the first three methodologies in detail. Methodologies 4 and 5 are discussed in Section 4 and Section 5, respectively.

#### 3.3.1. MIMIC-III Dataset 

We explored and selected the MIMIC-III dataset, and in this subsection we describe it in detail. In this study, we used this dataset for our model validation. 

##### Dataset Description 

The Medical Information Mart for Intensive Care (MIMIC-III) [57] is a publicly available clinical database maintained by the Massachusetts Institute of Technology (MIT) Laboratory. This database provides critical clinical data for over 40,000 patients admitted to intensive care units at the Beth Israel Deaconess Medical Center (BIDMC) in Boston, Massachusetts between 2001 and 2012. This database is populated with data collected during the patients’ routine hospital care during the mentioned period. The database includes patients’ clinical information, such as demographic information, laboratory test results, imaging reports, caregiver notes, medication, procedures, mortality, etc. 

MIMIC-III (v1.4) is a relational database consisting of 26 tables. All of these tables contain patients’ routine hospital care data. These tables are linked by unique identifiers such as patient-unique identity numbers. Therefore, the patients’ demographics and clinical information are spread across various tables. However, these tables are linked and can easily be accessed using the patient’s unique identity number. 

#### 3.3.2. Dataset Preprocessing

In this subsection, we describe the data extraction and cleaning methods that we employed to preprocess the MIMIC-III dataset in detail.

(a) Data Extraction

As mentioned above, the MIMIC-III (v1.4) database consists of 26 tables. Each table contains different types of patient data, such as the “Patient” table containing patient demographic information, the “Lab” table containing information on laboratory test results, etc. To prepare and test our model, we extracted data from the following three tables: Patient (patient demographic information), Lab (patient laboratory test results), and Admission Diagnosis (patients’ admitted diagnosis information). We selected data from these tables for our machine learning model because they provide the patients’ core clinical information in any healthcare unit. Therefore, we believe that this should be the most suitable dataset for our research.

(b) Data Cleaning

Data cleaning entails detecting and correcting any dataset’s incorrect and corrupt data elements. Every dataset has inaccurate elements, which must be corrected before further processing. Therefore, we cleaned our dataset before training our model on this dataset. 

The extracted data from the MIMIC-III database had many erroneous entries, such as patients’ date of birth, wrong format, missing or incorrect values in some attributes, missing labels, etc. We identified and handled the following two issues in the extracted data: 

First, we observed that there was inconsistency in the recording of some variables. For example, in the Patient table, the values stored in the DOB (date of birth) attribute are not in a single format. Similarly, the lab unit value was also missing for some records in the Lab table. Second, some variables had multiple values recorded at the same time for a single patient, such as lab values or units, etc. Similarly, in the Admission Diagnosis table, the diagnosis codes for some patients’ records were either missing, incorrect, or incomplete. Moreover, some records used the diagnosis codes from other coding systems. Therefore, we preferred handling these issues before processing and applying our machine learning model. The collected data were preprocessed for missing values, duplicate values, etc. For this purpose, we used the OpenRefine tool—an open-source desktop application used for data cleaning and transformation to other formats [58]. Furthermore, we also used manual techniques for data cleaning.

We addressed these issues by using the following procedures:

1. Handling inconsistency units: To deal with inconsistency, we searched the entire “Patient” table and addressed those entries whose format was different from the others. We manually updated these patients’ date of birth to other entries’ format to ensure that it was consistent with other entries in the same table. Similarly, we thoroughly accessed the “Lab” table entries and updated the missing entries. Furthermore, we searched the entire “Admission Diagnosis” table and replaced the diagnosis codes for some entries with the new coding system to ensure that they were consistent with other entries in the same table. We made these changes because they used two coding systems for some records. 

2. Handling multiple recordings at the same time: We checked every record and deleted duplicate entries. We kept the value that appeared first and discarded the later value. 

3. Handling missing values: We checked every record and updated the missing values. For this purpose, we inserted missing values to complete the records. Some examples include the Lab table’s missing entries, such as units of measure, diagnosis codes, etc. 

#### 3.3.3. Dataset Preparation

We needed the dataset to be in the HL7 v2 messages format for our framework’s validation, but the data that we retrieved from the MIMIC-III database were in a simple EHR format stored in the database tables. Therefore, first, we converted these data into the HL7 v2 message format to prepare the dataset.

##### EMR Data Conversion to an HL7 V2 Message Dataset 

The collected data were the clinical data used in the various information systems of the hospital. As mentioned above, these data were stored in simple database tables in an EMR format. We collected almost 53,410 records. Once the required dataset was ready, we decided to convert this dataset into the HL7 v2 message format. The task was tricky; unfortunately, no automatic support was available, such as any tool or plug-and-play facility to automatically convert any EHR clinical data stored in database tables or CSV files into the HL7 v2 message format. 

Therefore, we decided to develop a middleware engine to convert (i.e., map) the medical data stored in simple flat files—such as CSV files or database tables—into the HL7 v2 message format. This engine can take raw EMR data as inputs and produce HL7 v2 messages. It reads the data stored in a CSV file and automatically maps to an equivalent HL7 v2 message. We included a raw EMR dataset with a total of 53,410 records of patients’ demographic information, various lab test results from various types of labs, and diagnosis descriptions. Each data type was mapped to different HL7 v2 messages, because every HL7 v2 message has different segments and fields for each data element. Therefore, their mapping to HL7 v2 messages is also difficult. We used the Mirth engine tool for this data mapping—an open-source, cross-platform interface engine used in the healthcare industry for clinical data mapping and exchange purposes [59]. We created channels for each message and algorithmically mapped the EMR data to HL7 v2 message segments. 

**Figure 2:**
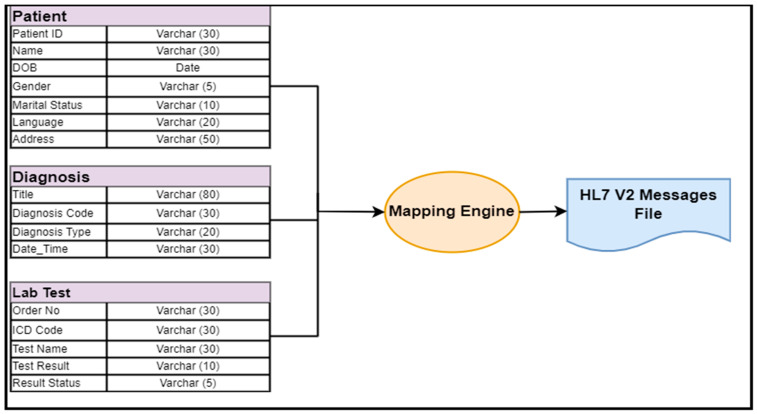
EMR-to-HL7 v2 data mapping process.

The mapping engine is based on various algorithms and can convert any EMR dataset into an HL7 v2 message dataset. The dynamic mapping engine automatically maps the data to any type of HL7 v2 message segment. It reads the data from the EMR dataset and converts them into HL7 v2 messages (one record per message). The resulting HL7 v2 message dataset is stored in a separate “text file”. This dataset was the input dataset for our framework. It is very difficult to display the complete picture of this dataset (text file). However, to give the reader a clear view of our dataset, a few messages of the resulting HL7 v2 dataset are summarized in Table 1. Additionally, Figure 2 shows the entire mapping process.

## 4. Methods

In this section, we describe the DBSCAN algorithm and develop and analyze various algorithms, data representation, processing, and the required outcomes in detail. 

### 4.1. Algorithms

We developed the model to generate the result once the dataset became ready. We planned to use the DBSCAN algorithm to develop our model. However, the DBSCAN algorithm could not operate on data stored in HL7 v2 message segments. Therefore, we performed a two-step process to develop our model. In the first step, we developed an algorithm to retrieve the data from the HL7 v2 dataset and convert them to a simple EMR format. In the second step, we exploited the DBSCAN algorithm to develop our model based on the dataset that we developed in step 1. 

Step 1: Conversion of the HL7 v2 message dataset to a simple EMR dataset

No common HL7 v2 message stores all types of clinical data. The clinical data were stored in different HL7 v2 messages, and every type of message had various segments and fields. For example, patient demographic information was stored in one type of message segment, lab test result information was stored in another type of message segment, etc. 

Therefore, our HL7 v2 message dataset had different types of messages, and each type of message had a different segment type. The data were stored in these message segments. Furthermore, the HL7 v2 messages in the dataset were stored in mixed form and in a random order. Therefore, retrieving the data from these various types of message segments was tricky, and there was a need for a dynamic and intelligent algorithm to recognize every type of message segment before data retrieval and storage in the resulting file. We developed the algorithm shown in Algorithm 1 to retrieve the data from the HL7 v2 message dataset and convert them to a simple EMR format. The algorithm read the data from the input dataset (HL7 v2 messages) one message at a time and converted them into the EMR data format. The resulting data were stored in a single CSV file in the order that they were retrieved from the HL7 v2 dataset. This was used as the input dataset for our machine learning model.
**Algorithm 1** Parse HL7 v2 messages 1: **Function** read_by_chunk (file) 2: Remove empty line from data file (if there is any)  3: Declared a variable name List  4: **for each** line of the file **do** 5:   **if** line is not newline **then** 6:    Remove/r from the line and appends to a List  7:   **else** 8:    Yield the concatenated List 9:   **end if**
  10: **end for**
  11: **return** List  12: **end function** **read_by_chunk function**  13: **Read data from HL7 v2 messages segments and fields**   14: **Function** init_dataframe (file)   15: var File = read_by_chunk (file)   16: **while** (!EOF) **do**  17: Read chunk from File   18: Run chunk through HL7 parser   19: extract data from each category   20: create dataframe from the data extracted  21: dataframe.to_csv () // appends dataframe to csv file (resulting file)  22: **end while**  23: **end function** **init_dataframe function**

The input data were stored in various HL7 v2 segments. Therefore, once they were converted to the structure format (i.e., CSV file), we again cleaned this dataset. We manually searched the entire file and removed some redundant information from this dataset before we developed our machine learning model. Once the dataset was clean and ready, we developed our machine learning model. 

It is very difficult to display a complete picture of the resulting dataset that was obtained. However, to give the reader a clear view of this dataset, we represent a small section of this dataset (with a general CSV file structure) as an example in Table 2.

Step 2: Using the DBSCAN algorithm to cluster the EMR dataset developed in step 1 

In this step, we exploited the DBSCAN algorithm to cluster the dataset that we developed in step 1. However, before using the DBSCAN algorithm for our model, we should discuss the DBSCAN algorithm to give the background of this algorithm to the reader. 

### 4.2. DBSCAN (Density-Based Spatial Clustering of Applications with Noise)

Clustering and cluster analysis [60,61] are widely used methods in data analysis, and their key function is to organize data in similar groups (i.e., clusters) based on similar types of data elements and objects, such that the data elements in one cluster are similar and are different from those in other clusters. Many clustering algorithms and techniques are available, and each has its merits and demerits. However, the density-based spatial clustering method is one of the most commonly used density-based clustering algorithms. It is the most efficient and effective algorithm for handling large datasets. 

After studying and analyzing numerous clustering algorithms [60], we decided to use the DBSCAN algorithm in this work for our data clustering because it is specially designed to discover the clusters and noises in large datasets. Some of the key reasons that we selected the DBSCAN algorithm are given below:

1. It works on global parameters and is not based on specific parameters.
2. The users do not need to predict or specify the number of clusters in advance; hence, it is more realistic than all other clustering algorithms.
3. It can identify outliers in the clusters, and the possibility of merging with other clusters exists if they are similar.
4. The selection of attributes is always open to improvement in terms of temporal and spatial complexity.

The first three points (1, 2, and 3) match our requirements, and we needed an algorithm that had these capabilities. Therefore, we selected this algorithm for this research work. 

We had a large clinical dataset, and our main objective was to train our model for clustering based on variables (i.e., attributes) mentioned in the input dataset. Moreover, we did not need to identify the number of output clusters, because the model would be responsible for doing it automatically. More importantly, the model could work on any dataset, as opposed to just our specific dataset, i.e., if we changed the input dataset, the model or algorithm would still work without any modification. Therefore, it should work on global parameters and be common for all clusters. 

For this reason, we would have to identify each cluster’s appropriate Eps and MinPts parameters and at least one point from the respective cluster. Upon identifying these points, we would be able to fetch all points that are accessible from the given points using the appropriate parameters. This process was extremely tricky, and getting the information in advance for the clusters of the input dataset was difficult. However, a simple heuristic algorithm (DBSCAN) was available to determine the thinnest parameters Eps and MinPts in the input dataset. Therefore, the DBSCAN algorithm uses global values for Eps and MinPts, meaning the same values for all clusters. 

**Figure 3:**
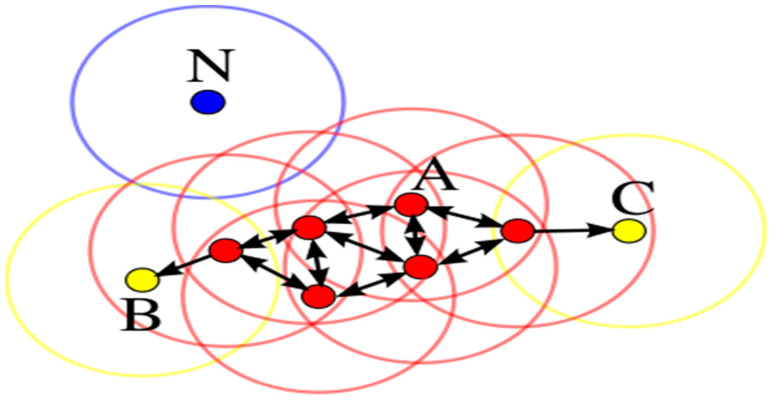
Red points are core points; points B and C in yellow are density-reachable, and point N in blue is a form of noise [62].

Before discussing the DBSCAN algorithm in detail, we need to clarify some concepts and definitions that are directly and indirectly related to DBSCAN. They are explained as follows:

1. **Cluster:** In a database with *N* given data points, e.g., *D* = {p1, p2, p3,..., p*n*,}, the process of splitting database *D* into smaller parts that follow similar formats, e.g., *C* = {c1, c2,..., c*i*}, is called clustering. There are a total of *Cj* clusters, where *Cj* ≤ *D* (*j* = 1, 2, 3. . . *i*).
2. **MinPts:** The minimum points needed to form a dense region in a database are called MinPts. In Figure 3, there are a total of 4 MinPts.
3. **Eps:** The Eps defines the radius of the neighborhood around a point “x”. This is called the ϵ-neighborhood of x.
4. **Neighborhood:** A distance function for any two points *p* and *q* denoting dists (*p*, *q*) is called a neighborhood.
5. **Eps-neighborhood:** The Eps-neighborhood (threshold distance) of a point *p* is defined by [*q* ∈ *D* | dist (*p*, *q*) ≤ Eps].
6. **Core point:** A point *p* is a core point if at least MinPts points are within a distance ∈ of it, and those points are said to be directly reachable from *p*. In other words, a core point/object is a point in its neighborhood of a given radius (Eps) that has to contain at least a minimum number (MinPts) of other points. In Figure 3, point A and all other red color points are called core points/objects, while points B and C are not core objects.
7. **Border objects:** An object *p* is called a border object if it is not a core object but a density accessible from another core object. In Figure 3, the green circle is called a border object.
8. **Noise:** The points that are not reachable from any other points are called noise and are assigned to a cluster. In Figure 3, point *N* in blue is called noise (noise = {*p* ∈ *D* | ∀: *p* ∉ *Ci*}).

### 4.3. Algorithm Development

Our prime research objective was to identify proper clusters for different types of clinical datasets. Therefore, we decided to design and develop a clustering algorithm that works on global input parameters and automatically clusters its data into various clusters. For this purpose, we used the DBSCAN algorithm. The DBSCAN algorithm requires two parameters mentioned in [63]:

1. Eps is a radius representing a neighborhood’s attributes around a particular point (say x).
2. MinPts represents the minimum number of neighbors within the “Eps” radius.

Given a set of points (dataset) for clustering, DBSCAN uses the neighborhood radius (Eps) and the minimum number of points in the neighborhood (MinPts) to classify the points into border points in the clusters, core points in the clusters, and outliers. To identify a cluster, DBSCAN starts with random points, e.g., p, and determines all density-accessible points from p with respect to Eps and MinPts. If p is a core point (i.e., N_Eps_ (p) > MinPts), then this creates a cluster with respect to Eps. Moreover, p and all points that are density-accessible are gathered in one cluster. However, if this p is a border point and no points are density-accessible from p, then the DBSCAN algorithm visits the next point of the dataset. This algorithm is simple and efficiently generates the result, but it becomes complicated when working with global values for MinPts and Eps. 

We needed to use global values for MinPts and Eps, in which if two clusters of different densities are close to one another, DBSCAN may merge them into a single cluster. Therefore, we had to look for other scenarios as well. For example, if the distance between two sets of points (e.g., X1 and X2) is defined as D (X1, X2) = min {D (i, j), where i ∈ X1, j ∈ X2}, then two sets of points with at least the density of the thinnest cluster will be set apart from one another in that situation if the distance between the two sets is larger than the Eps value. Otherwise, a recursive call of DBSCAN is needed for the detected clusters with a higher value for MinPts. 

Here, we represent a simplified version of the DBSCAN algorithm in Algorithm 2 that we used for data clustering in our model. However, the data types and other detailed information about the clusters are not shown here.
**Algorithm 2** Machine learning model  1: Input 2:    Set Of Points: The input whole dataset/discovered cluster from the previous run 3:    Eps & MinPts: The global density parameters  4:    The MinPts is set to default values (5 in our case)  5:    Eps is specified by the user (3 in our case) 6: Output  7:    Clusters with their core objects and noise points as *C* = {*c*1, *c*2,...,} 8:    Various statistics about the dataset, such as gender details, lab test descriptions,      diagnosis Information, etc. 9: Method  10: **Function** Cluster (SetOfPoints, Eps)  11: var List = Read (Dataset) ** Read the dataset and stored in List variable**  12: **Encode the List using one hot encoding and set the encoder to auto**  13: var encode = OneHotEncoder (categories=’auto’)   14: var encode_data = encode.fit.transfered.List [1 :]) .toArray ()   15: cluster = DBSCAN (encode_data, eps=eps) ** Clusters the encoded data**  16: Cluster_Split (cluster.labels, List) ** Split the input file according to the clusters*   17: Display all clusters **Display all clusters**  18: Calculate_Statistics () **Calculate various statistics**  19: **end function** **Cluster function**   20: **The most important function used in this algorithm to split the main cluster to various clus   ters is Cluster_Split which is presented below**   21: **Function** Cluster_Split (clusterLabels, InputFileList)  22: clusters = [1, 2, 3, 4]  23: header = InputFileList[ 0 ] **Ignore the 1^st^ row of input file, i.e., attributes names in input file**  24: InputFileList = InputFileList [1] **Read data from 2^nd^ row of input file**  25: **for each** line of the clusterLabels **do** **From 1 to total no of records in input file**  26:  **if** line not in cluster **then**
  27:   clusters.append(line)  28:  **end if**
  29: **end for**
  30: **for** i = 1 to range (len (clusters)) do **within the cluster list**  31:   with open (’cluster ’+str (i) +’.csv’, ’w’, newline=’’) as file **Create file for each cluster**  32:   writer = csv.writer(file) ** initiate the writer for file**  33:   writer.writerow(header) **Write the header to the cluster file**  34:   **for** j = 0 to inputfilesize 10000 in range (len(clusterLabels)) **do**  35:    **if** clusterLabels[ j ] == i **then**  36:     writer.writerow(inputFileList[j]) //Write the data to the cluster   37:    **end if**
  38:   **end for**
  39: **end for**
  40: **end function** **Cluster_Split function**  41: **In this algorithm the Calculate_Statistics function is used to calculate various statistics of our    dataset, as described below**   42: **Function** Calculate_Statistics ()  43: Open clusters files (csv) directory **Open directory that stored all clusters files**  44: Files_Count = Read (files from directory)   45: ** Run the loops through all .csv files in the directory**  46: **while** (!EOF) **do**  **Read the Files_Count files**   47:   Get cluster file column(s)   48:   Calculate the number of entries in each file   49:   Calculate each cluster percentage   50:   Creates a DataFrame of all percentages and column entries  51:   Outputs a .csv file with the DataFrame  52: **end while**
  53: **end function** **Calulate_Statistics function**

The dataset was either the entire dataset or a discovered cluster from the previous run. Eps and MinPts were the global density parameters that were set manually. First, we read the input dataset and stored it in the variable (List). The next step was to encode the dataset using a one-hot encoder. When the encoding dataset was ready, we clustered this encoding dataset, which was a two-step process. In the first step, the whole dataset was converted to one cluster, and in the second step this single cluster was further split into more clusters.

### 4.4. Algorithms Development and Analysis

We were particularly interested in developing a dynamic algorithm that could be run on any clinical dataset used in any healthcare unit, rather than relying on one particular dataset. Moreover, it had to be an unsupervised machine learning clustering algorithm capable of clustering our dataset without any manual interaction. Therefore, we searched for an unsupervised machine learning clustering algorithm that did not need any specific input parameters, and without specifying the output clusters’ details. We believed that when the algorithm worked without any input parameters, we could run it on any type of clinical dataset regardless of the number and type of input variables (i.e., parameters). Therefore, we exploited the density-based spatial clustering of applications with noise (DBSCAN) [64] unsupervised machine learning clustering algorithm. The advantage of this algorithm is that it works on any type of heterogeneous dataset without any manual interaction. This algorithm can process any volume of data and filter them to unlimited clusters depending on the variable types mentioned in the input datasets.

Because the model under discussion only splits the input dataset into various clusters and cannot give any useful information about the variables (data) in the resulting clusters, the resulting clusters do not fit directly for further processing. To make the resulting clusters more meaningful and fit for further processing, we manually processed each resulting cluster, carefully observed each data element and its relationship with other elements in the cluster, and assigned appropriate labels for each cluster. Because we planned to map these data to FHIR resources in the future, we manually labeled each cluster with their title, e.g., the cluster-stored patient demographic information was labeled with “Patient Cluster”, the laboratory test results cluster was labeled with “Lab Result Cluster“, the diagnosis cluster was labeled with “Diagnosis Cluster”, etc. The entire process was a two-step process in which we automatically divided the dataset into various clusters and then manually labeled each cluster.

### 4.5. Data Representation and Processing

We were particularly interested in developing a data structure that could work for any FHIR resources in the future. We were excited to develop and train our model on any clinical dataset, as opposed to on a particular dataset. Furthermore, the resulting data structure could be a map to the FHIR resources. Therefore, we developed a single data structure that could be mapped to various FHIR resources rather than mapping only to one or two specific resources. Additionally, the model could be trained on any dataset rather than on a particular dataset. Our model is so general and dynamic that it can process and generate accurate results on any dataset.

### 4.6. Required Outcomes

We were interested in understanding whether clustering techniques could be used to produce the patient clinical data (HL7 v2 dataset) used in any healthcare organization in a structure that could be directly mapped to the FHIR resources. Furthermore, we were also interested in whether a machine learning model could be trained to parse a variety of clinical data from information systems to different clusters and produce valid clusters of input data. Therefore, we selected the input data from divergent health information systems, such as admission, laboratory, and diagnosis information systems (diagnosis classification systems). The expected output clusters would also be from the same domain. Therefore, the expected outcome clusters comprised the patients’ demographic information, laboratory test result details, and diagnosis description.

As a result, our machine learning model clustered the clinical data into various clusters according to their types and the correlation of input variables. Since the input data were the clinical data, the resulting cluster’s data would be able to map to the FHIR resources. Moreover, we also needed some statistics about this dataset to perform some data analytics on this clinical dataset in the future, which constituted another objective of this research work. However, we do not discuss any data analytics here. Therefore, applying or using data analytics is beyond the scope of this paper.

## 5. Experiments, Evaluation Measures, and Results

In this section, we discuss the experiments and results in detail. We performed various experiments and obtained the following results:

### 5.1. Experimental Setup

In this subsection, we discuss the experiments that we performed during our model’s implementation and the validation of data clustering. The program was written in the Python programming language, and the experiment was carried out on a machine with a 32-core processor, 256 GB of RAM, and the Windows 10 OS, where the software stack consisted of scikit-learn with an ANN backend. Moreover, we also used the Mirth engine tool for some experiments.

We performed our experiments in three stages: We experimented on the EMR dataset in the first stage and produced the HL7 v2 dataset. In this stage, the results were based on a simple EMR dataset, producing a list of HL7 v2 messages. This result was stored in a simple “text file”, and this file was used as the input dataset for our machine learning model. A sample of this file is shown in Table 1. For this experiment and the algorithms’ implementation, we used the Mirth engine tool.

The machine learning DBCAN algorithm cannot operate on the HL7 v2 message dataset. Therefore, in stage 2, we performed a second experiment on the dataset that we produced in stage 1. We used the algorithm mentioned in Algorithm 1, and the experiment produced the results shown in Table 2. The data mentioned in this table were used as the input dataset for the machine learning model.

In stage 3, we performed an experiment on our machine learning model. We tested the model and generated the results. The experimental results were based on the best hyperparameters specified as input parameters. We set the hyperparameters (i.e., Eps = 3, MinPts = Default value (5)) and dataset. The reason for setting only two input parameters (i.e., dataset and Eps) and leaving all others as default values was to make our model algorithm free-handed so that it could run on any dataset—as opposed to working only on this dataset—without any further modification or requesting any input variables. This capability is the key advantage of our model, because we do not want to restrict our model to a particular dataset. As we ran our model, it successfully categorized the input data into various clusters. Our model could categorize any clinical dataset into various clusters regardless of the input variables. Therefore, we changed the datasets a couple of times and ran the algorithm with the new datasets. Each time, the model generated the result successfully. For example, initially, we ran the model on our dataset, and it categorized the dataset into three clusters. We changed the dataset and ran the model, and it displayed the results in four different clusters (i.e., classes).

Similarly, the model categorized the input dataset into six clusters when we changed the dataset for the third time and ran the model. These results show the divergent functionalities of our model. However, this was only for model validation and testing purposes. In the end, we focused on our original dataset and its results. Therefore, we included the early results in this article and discarded the later ones.

### 5.2. Model Evaluation Measures

Accuracy, sensitivity, and specificity matrices were used in this experiment to evaluate the performance of our model. A total of 42,728 instances were used for the training dataset. We used the precision, recall, and F1-score measures to evaluate the accuracy of our model. To calculate all of these measures, a confusion matrix is needed, which consists of the true positive (TP), false positive (FP), true negative (TN), and false negative (FN). The formulation of the measures is given in Table 3. We performed experiments on three different datasets: first, for the main dataset (42,728 training instances); second, for the lab cluster (10,995 training instances); and third, for the diagnosis cluster (28,900 training instances). Therefore, we used all four measures (accuracy, precision, recall, and F1-score) to evaluate the model’s accuracy and performance for all three results. The experiential measures were calculated using performance factors such as clustering accuracy and execution time.

### 5.3. Results

We included hospital care records for a total of 53,410 patients, involving 13,747 (25.74%) from patient admission systems (patient demographic records), 3517 (6.58%) patient laboratory test results from laboratory information systems, and 36,146 (67.67%) diagnostic details from diagnosis classification systems. We trained our model so that it could produce three different clusters. However, this was only our expectation, and we never forced the model for such a result. We allowed the model to run openly, and in the end the result was the same as we had expected. The model categorized our dataset into three different clusters without any labels. Our principal objective was to map data of the resulting clusters to FHIR resources. Therefore, we manually labeled each cluster with an appropriate title after obtaining the clustering results. 

#### 5.3.1. Clustering HL7 V2 Dataset

We applied the machine learning DBSCAN algorithm on the HL7 v2 dataset (42,728 instances of training data), in which the clinical data were clustered in three classes according to the input variables mentioned in each class, e.g., patient demographic information, laboratory test result information, and medical diagnosis details. Our model results for the HL7 v2 dataset were excellent, and the clustering accuracy for the HL7 v2 dataset was 99.95%. The accuracy measure and the execution time for the DBSCAN algorithm in this result are shown in Table 4.

**Figure 4:**
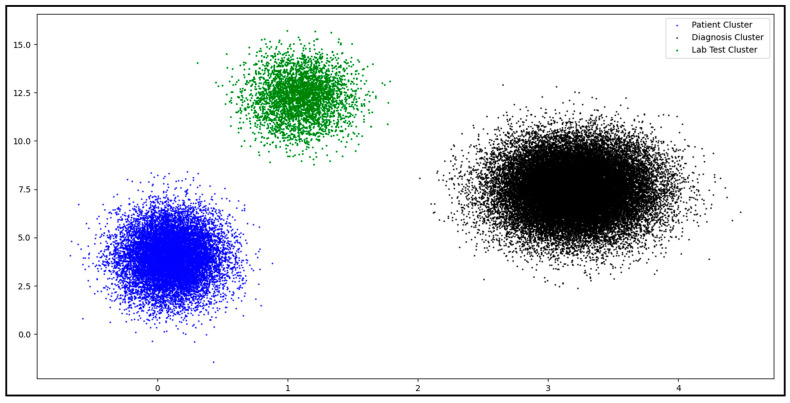
HL7 v2 dataset clustering result.

Our results reveal that DBSCAN exhibited the best performance in terms of unlimited variables in the input dataset. The clustering results are summarized in Figure 4**,** and the DBSCAN model performed better. There are many possible reasons for this high confidence in separating the data elements. One could be the high number of records in the input dataset. Machine learning usually requires a high number of records for generalization, indicating that this model is suitable for large datasets.

#### 5.3.2. Dataset Subclustering

The model successfully clustered the HL7 v2 dataset by producing three appropriate clusters. We already planned to cluster the dataset properly. Therefore, we further tested our model on individual clusters and checked the results. The results of one cluster (Patient) remained unchanged. This showed that further clustering was not possible for this cluster, and all variables in this cluster were homogeneous. However, the results of the other two clusters (Lab Test and Diagnosis) changed, and further classifications of these clusters arose. These new results were due to domain value variations in some attributes, and the model further classified the data in these two clusters to further clusters. Based on our experiment, the model further classified these clusters into various clusters. Their details are discussed in the following sections:

(a) Lab Test Clustering Classification

**Figure 5:**
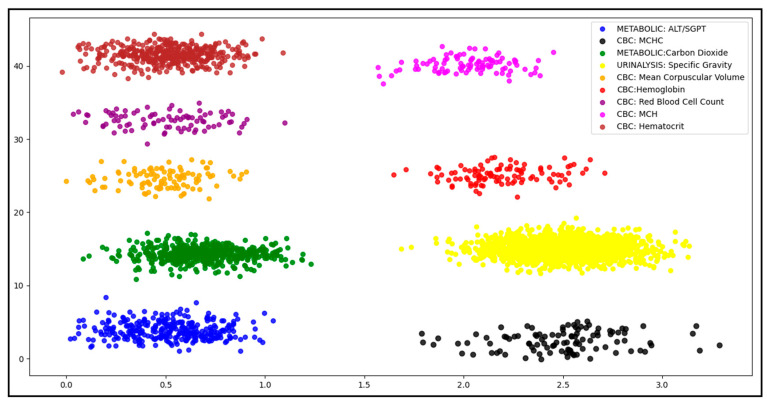
Lab test subclusters.

In the resulting cluster (Lab Test), the test name domain values were heterogeneous, and further classifications were possible based on the test name. We further tested our model on this dataset (Lab Test cluster), and the model classified it further into nine different clusters based on the test name attribute. In our dataset, we had nine test types, each with a different title; therefore, the model categorized this cluster into nine different categories. The results are summarized and presented in Figure 5.

Our model’s result for the Lab Test dataset classification was also effective, and the clustering accuracy for the Lab Test dataset was 99.63%. The accuracy measure and the execution time for the DBSCAN algorithm in this result are presented in Table 5.

(b) Diagnosis Clustering Classification

**Figure 6:**
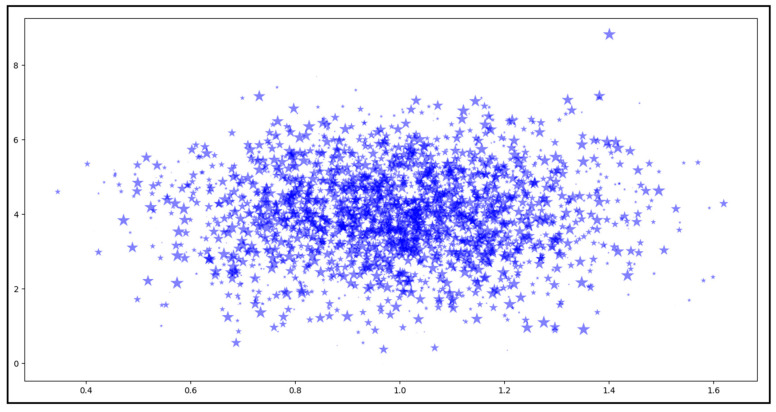
Subclustering classification (Diagnosis cluster).

In the resulting cluster (Diagnosis), the diagnosis code and descriptions had numerous domain value categories. Therefore, the possibility of further classifications existed, for which we trained our model. We trained our model on this dataset (Diagnosis cluster), and the model classified this cluster into a further 2854 clusters based on diagnosis codes. We therefore believed that 2854 different types of diagnosis code existed in our dataset, and the model was trained to classify them. Our model successfully categorized this dataset into various clusters and generated the results. To show the results of all 2854 clusters is quite difficult. However, to give the reader a brief idea about this classification, in Figure 6, we show only the clusters, where each STAR (*) represents one cluster. The size of each STAR (*) represents the number of records in that cluster.

Our model results for the Diagnosis dataset’s classification were also accurate, and the clustering accuracy for the Diagnosis dataset was 98.80%. The accuracy measure and the execution time for the DBSCAN algorithm in this result are presented in Table 6.

### 5.4. Cohort Analysis

We planned to perform various data analytics on this dataset and identify the details of individual data elements in our dataset, such as the number of patients by gender, specific race, marital status, etc. Therefore, we analyzed the entire dataset and performed some data analytics to calculate these statistics. The patient demographics and utilization characteristics are summarized in Table 7. Similarly, the dataset included various test results and their description. The details of the individual test statistics are summarized in Table 8.

We also wanted to understand the various diagnosis descriptions that we identified in the dataset. We had 2854 different diagnosis descriptions in our dataset, and we calculated the statistics of all diagnosis types. However, it was extremely difficult to represent the complete set of all of these diagnosis statistics. Therefore, Table 9 shows a subset of the diagnosis information in our dataset. The ICD-10 (International Classification of Diseases, 10th Revision) was used for diagnosis classification in this dataset.

## 6. Conclusions

Clustering algorithms are attractive for classifying information in a particular dataset. However, many well-known algorithms suffer from serious drawbacks when applied to large datasets. The majority of algorithms work based on user-defined input parameters and output cluster specifications. Therefore, developing a generalized algorithm that works on any dataset is challenging. However, we developed an algorithm that did not rely on specific input parameters and did not need to specify any output cluster details. In this article, we clustered our dataset using the DBSCAN algorithm, which relies on the density-based notation of clusters. The advantage of this algorithm is that it uses global parameters and supports the user in determining appropriate values for them. We also performed various operations on this dataset and generated different statistics. These statistics are very useful and could be used to perform various data analytics on this dataset in the future.

We presented the evaluation results of the machine learning model and applied it using the publicly available MIMIC-III. We developed our heterogeneous HL7 v2 message dataset using the clinical data from the MIMIC-III dataset. Once the HL7 v2 message dataset was ready, we applied the most popular and sophisticated unsupervised machine learning approach to this dataset and classified it into various clusters. We found conclusively that the machine learning model showed better results and categorized the clinical dataset into various clusters that could be further mapped directly to FHIR resources. Additionally, this approach could produce better results when many raw clinical data are used as inputs to produce the clustering results. We also found that in some specific cases, further classification of the resulting clusters could be possible after clustering the base dataset.

Our prime objective was to develop a data structure that could be easily mapped to FHIR resources. Needless to say, we successfully achieved this aim. In the future, we plan to extend this work and develop a dynamic algorithm to map these data clusters to FHIR resources. We believe that each data cluster could be a map to a single FHIR resource. Additionally, the generated statistics could be helpful in performing a variety of data analytics on this dataset.

## Figures and Tables

**Table 1:** Sample messages of the input dataset (HL7 v2).

MSH|^~\&|MIRTH_CONNECT|Brown-HOSPITAL|RECEIVINGAPP|Blue-HOSPITAL|20211002035037||ADT^A04^ADT_A01|11742|T|2.5||||||||| EVN|A01|20211002035037||||| PID|||639B5FFC-B9E3-4C8D-8F62-BABE52E4F3B9||Irma^Cook||19920308|F|||3789 Dancing Dove Lane^^New York^New York^10013^US||347-594-6300^PRN^^~IrmaSCook@fleckens.hu^NET^^|||||||||||||||||||||||||| PV1|1|I|4E^IS^Lab^Brown Hospital||||Dr.John||||||||||||||||||||||||||||||||||||||||||||| AL1|1||PENICILLIN||PRODUCES HIVES~RASH|| DG1||I10|S82^ANKLE FRACTURE^I10|ANKLE FRACTURE|||||||||||||||||||||||||||||||| MSH|^~\&|SendingLabX-RayLab|Brown-HOSPITAL|RECEIVINGLab:LISLab|Blue-HOSPITAL|20211002043236||ORU^R01^ORU_R01|4084|T|2.5||||||||| PID|||639B5FFC-B9E3-4C8D-8F62-BABE52E4F3B9||Irma^Cook||19920308|F|||3789 Dancing Dove Lane^^New York^New York^10013^US||347-594-6300^PRN^^~IrmaSCook@fleckens.hu^NET^^|||||||||||||||||||||||||| ORC|NW|987654^EPIC|76543^EPC||Final||^^^20140418170014^^^^||20140418173314|1148^PATTERSON^JAMES^^^^||1173^MATTHEWS^JAMES^A^^^|1133^^^222^^^^^|(618)222-1122|| OBR|1||Order_NO_01|10180-8^METABOLIC:ALT/SGPT^LN||||20211002043236||||||||D001^DR.^Rivera^Charles||||||||||||||||||||||||||||||||| OBX|1|SN|14647-2^METABOLIC: CHLORIDE^I10 ||101.8|mmol/L|||||F|||199203141030|||||| OBX|2|NM|13457-7^Fasting Blood Glucose^I10||178|mg/dl|70-100 mg/dl|H|||F| ||199707281152||||||

**Table 2:** Input dataset sample.

PID	DOB	Sex	Race	Primary Language	Marital Status	Test Name	Test Result	Unit of Measure	Collection Date and Time	Visit No	Diagnosis Code	Diagnosis Description
2F78B50C-103A-427A-A75C-29A789501678	197512101450	Male	Asian	English	Married							
915BC24E-8C44-4D33-A386-CEA965B83F32						METABOLIC: ALT/SGPT	17.8	U/L	194611261152			
ED06DB81-3D87-4206-9A5B-65AD89E65267										1	D20.0	Benign neoplasm of soft tissue of the retroperitoneum
639B5FFC-B9E3-4C8D-8F62-BABE52E4F3B9						METABOLIC: BILI TOTAL	1	mg/dL	194902161152			
AC6F9199-ACF0-4A45-924B-ECE5D3843A56										1	M63.872	Disorders of muscle in diseases classified elsewhere—left ankle and foot
108B06ED-347B-496F-AB3E-3D0F42906C69	197512121450	Female	White	English	Married							
A6ADB61A-6993-4D14-93C9-B3D46014C114						CBC: WHITE BLOOD CELL COUNT	8.6	k/cumm	196212231152			
98CC2D50-B848-48D1-8C63-2A7C2761FE59										1	D43.3	Neoplasm of uncertain behavior of the cranial nerves
F4A580D8-E8C0-4432-B485-E1EED456118B	197512191450	Female	White	English	Separated							
3AB69ECE-65F4-4D04-9E87-54E73C2DB4A8										1	C40.31	Malignant neoplasm of short bones of the right lower limb
4DD3728F-0C48-43B7-B488-421C85A5E931						METABOLIC: CHLORIDE	98	mmol/L	199603211152			
DF598997-15DE-4658-A4D4-48A4ABA08179	197512301450	Male	White	Spanish	Single							
43556DC2-BCFC-45A8-84C3-1D3E4A11B02F										1	G31.83	Dementia with Lewy bodies
ED64D34E-8945-4073-871D-4B9AC166B0CD						CBC: MCHC	36.1	g/dL	203909191152			
32EB8347-A355-4E49-A551-41C4DE28436A		Male	African American	Unknown	Divorced							

**Table 3:** Measures used for model evaluation.

Matric	Formula
Precision (P)	TP/(TP + FP)
Recall (R)	TP/(TP + FN)
F1-Score	2 × (P×R)/(P + R)
Accuracy	(TP + TN)/(TP + TN + FP + FN)

**Table 4:** Algorithm performance factors for main clusters.

No. of Clusters	Cluster Statistics			Execution Time in Seconds	Unclustered Instances	Accuracy (%)	Precision (%)	Recall (%)	F1-Score (%)
3	Clusters	No. of instances	Instances (%)	51.4	27	99.95	98.41	99.31	98.93
	Cluster 0	3515	6.58						
	Cluster 1	13743	25.74						
	Cluster 2	36125	67.67						

**Table 5:** Algorithm performance factors for Lab Test subclustering.

No. of Clusters	Cluster Statistics			Execution Time in Seconds	Unclustered Instances	Accuracy (%)	Precision (%)	Recall (%)	F1-Score (%)
9	Clusters	No. of instances	Instances (%)	0.4	13	99.63	99.12	98.91	98.72
	Cluster 0	390	11.13						
	Cluster 1	289	8.24						
	Cluster 2	105	2.99						
	Cluster 3	527	15.03						
	Cluster 4	1810	51.65						
	Cluster 5	100	2.85						
	Cluster 6	104	2.96						
	Cluster 7	87	2.38						
	Cluster 8	92	2.62						

**Table 6:** Algorithm performance factors for Diagnosis subclustering.

No. of Clusters	Cluster Statistics			Execution Time in Seconds	Unclustered Instances	Accuracy (%)	Precision (%)	Recall (%)	F1-Score (%)
2854	Clusters	No. of instances	Instances (%)	12.8	433	98.80	98.31	97.91	98.17
	Cluster 0	7	0.0192						
	Cluster 1	12	0.0332						
	Cluster 2	19	0.0526						
	Cluster 3	11	0.0304						
	Cluster 4	14	0.0387						
	Cluster 5	19	0.0526						
	Cluster 6	17	0.0470						
	Cluster 7	10	0.0277						
	Cluster 8	18	0.0498						
	Cluster 9	12	0.0332						
	Cluster 10	16	0.0443						
	Cluster 11	13	0.0359						
	………	………	………						
	………	………	………						
	Cluster 2854	2714	7.5084						

**Table 7:** Summary of patients’ demographic information.

Gender	Total	Gender (%)	Marital Status	Total	Marital Status (%)	Race	Total	Race Percentage (%)
Male	6609	48.08291	Single	4356	31.6841	White	6726	48.9252
Female	7136	51.9179	Married	6203	45.1190	African American	2059	14.9768
			Divorced	1552	11.2882	Asian	3172	23.0722
			Separated	685	4.9821	Unknown	1788	13.0051
			Widowed	133	0.9672			
			Unknown	816	5.9353			

**Table 8:** Summary of patients’ lab test results.

No.	Test Name	No. of Tests	Tests Percentage (%)
1	CBC: Hematocrit/RDW/Absolute Neutrophils/Absolute Lymphocytes	394	11.2027
2	METABOLIC: ALT/SGPT/ALK PHOS	289	8.2172
3	CBC: MCHC	105	2.9854
4	METABOLIC: Carbon Dioxide/Anion Gap/Sodium/Chloride/Potassium	527	14.9843
5	CBC: Mean corpuscular volume	100	2.8433
6	CBC: Hemoglobin METABOLIC: Albumin/Total protein	104	2.9570
7	CBC: Red blood cell count	87	2.4736
8	CBC: MCH	95	2.7011
9	URINALYSIS: Specific Gravity/PH	1817	51.5311

**Table 9:** Summary of various diagnostic information.

No.	Diagnosis Code	Diagnosis Description	No. of Diagnosis	Diagnosis Percentage (%)
1	F98.21	Rumination disorder of infancy	7	0.0192
2	G31.83	Dementia with Lewy bodies	12	0.0332
3	O9A.12	Malignant neoplasm complicating childbirth	19	0.0526
4	D89.81	Malignant neoplasm of the bladder neck	11	0.0304
5	M84.561	Pathological fracture in neoplastic disease, right tibia	14	0.0387
6	G52.3	Disorders of the hypoglossal nerve	19	0.0526
7	C40.31	Malignant neoplasm of the short bones of the right lower limb	17	0.0470
8	C67.5	Malignant neoplasm of the bladder neck	10	0.0277
9	O98.612	Protozoal diseases complicating pregnancy	18	0.0498
10	Z13.85	Encounter for screening for nervous system disorders	12	0.0332
11	Y71	Cardiovascular devices associated with adverse incidents	16	0.0443
12	C79.82	Secondary malignant neoplasm of the genital organs	13	0.0359

## Data Availability

The datasets used or analyzed in this study are available from the corresponding author upon reasonable request.
